# The Effect of Match Schedule on Accelerometry-Derived Exercise Dose during Training Sessions throughout a Competitive Basketball Season

**DOI:** 10.3390/sports6030069

**Published:** 2018-07-23

**Authors:** Craig Staunton, Daniel Wundersitz, Brett Gordon, Edhem Custovic, Jonathan Stanger, Michael Kingsley

**Affiliations:** 1 Exercise Physiology, La Trobe Rural Health School, La Trobe University, Bundoora 3086, Australia; C.Staunton@latrobe.edu.au (C.S.); D.Wundersitz@latrobe.edu.au (D.W.); B.Gordon@latrobe.edu.au (B.G.); 2 Computer and Mathematical Sciences, La Trobe University, Bundoora 3086, Australia; E.Custovic@latrobe.edu.au (E.C.); J.Stanger@latrobe.edu.au (J.S.)

**Keywords:** female, training load, monitoring, accelerometer, workloads

## Abstract

Accelerometry-derived exercise dose (intensity × duration) was assessed throughout a competitive basketball season. Nine elite basketballers wore accelerometers during a Yo-Yo intermittent recovery test (Yo-Yo-IR1) and during three two-week blocks of training that represented phases of the season defined as easy, medium, and hard based on difficulty of match schedule. Exercise dose was determined using accumulated impulse (accelerometry-derived average net force × duration). Relative exercise intensity was quantified using linear relationships between average net force and oxygen consumption during the Yo-Yo-IR1. Time spent in different intensity zones was computed. Influences of match schedule difficulty and playing position were evaluated. Exercise dose reduced for recovery and pre-match tapering sessions during the medium match schedule. Exercise dose did not vary during the hard match schedule. Exercise dose was not different between playing positions. The majority of activity during training was spent performing sedentary behaviour or very light intensity activity (64.3 ± 6.1%). Front-court players performed a greater proportion of very light intensity activity (mean difference: 6.8 ± 2.8%), whereas back-court players performed more supramaximal intensity activity (mean difference: 4.5 ± 1.0%). No positional differences existed in the proportion of time in all other intensity zones. Objective evaluation of exercise dose might allow coaches to better prescribe and monitor the demands of basketball training.

## 1. Introduction

Training sessions contribute substantially to the total volume of exercise that basketball players receive (exercise dose; product of exercise intensity and duration) during the competitive season [1]. Although the exercise dose during basketball match-play has been extensively examined [2,3,4,5,6,7], the exercise dose associated with training sessions remains largely unreported [8]. Only one study to date has investigated the exercise dose received by players during the in-season phase of a basketball training program [1]. The results from this study showed that match schedule (i.e., no match, one match, or two matches per week) influences the exercise dose received by players [1]. However, these data were collected from only one two-week block during the in-season phase of competition. Previous research has identified fluctuations in exercise intensity during different phases of basketball pre-season training [8]. Therefore, it is plausible that the exercise dose received by players fluctuates throughout different phases of a competitive basketball season. However, no research to date has investigated the exercise dose received by players throughout multiple phases of a competitive basketball season.

The exercise dose and intensity of activity during basketball match-play and training are most commonly quantified using time-motion analyses and physiological data [2,3,6,9,10,11,12]. For example, movement speeds (derived from time-motion analyses) and heart rate responses are often used to measure exercise intensity. While time-motion analyses can be used to assess movement patterns undertaken and physiological responses provide a measure of average exercise intensity, the highly intermittent pattern of exercise and frequent vertical efforts make these methods inappropriate to quantify exercise dose during basketball match-play and training. In support of this statement, time-motion analyses underestimate the external demands of basketball-specific movements (e.g., jumping, shuffling, changes of direction) [13] and physiological analyses are associated with delays in responsiveness due to cardiorespiratory lag [14]. Thus, these techniques are incapable of accurately quantifying brief bouts of supramaximal intensity exercise and rapid changes in movements that occur frequently in basketball [2,3,6]. 

With the aim to circumvent the aforementioned limitations that are associated with other measurement systems, wearable accelerometers have emerged as an alternative method to quantify exercise dose during basketball. Accelerometers have high data acquisition rates and can measure activity in three planes of motion, making this measurement technique well-suited to quantifying the exercise dose and intensity in intermittent sports, such as basketball.

Average net force (AvF_Net_) is an accelerometry-derived measure of exercise intensity, with confirmed construct validity in basketball [13]. Strong relationships between accelerometry-derived metrics and oxygen consumption ($\dot{V}$O_2_) have been previously identified [7,15,16], exemplifying that accelerometers can be used to estimate relative exercise intensity. Additionally, supramaximal intensity exercise can be estimated from extrapolation of individual linear relationships between running speed and oxygen consumption [17,18,19]. Therefore, AvF_Net_ offers a measurement technique that is well-suited to calculate relative exercise intensity during intermittent sports, including the measurement of supramaximal intensity efforts. In addition to quantifying exercise intensity, the product of AvF_Net_ and exercise duration (Impulse) can be used to quantify exercise dose. Consequently, accelerometery-derived AvF_Net_ can provide a suitable method to quantify the relative exercise intensity completed by players during basketball training sessions, which could help coaches to prescribe more match-specific training and execute periodised training plans. 

The aim of this study was to use accelerometry-derived AvF_Net_ and accumulated impulse to assess relative exercise intensity and exercise dose during training sessions completed at different phases of a competitive basketball season.

## 2. Materials and Methods

### 2.1. Participants

Nine professional players (27 ± 5 years, 182 ± 8 cm, 81 ± 12 kg) from a basketball team competing in the Australian Women’s National Basketball League (WNBL) participated in this study. All players provided informed written consent and completed the requirements of this study. Ethical approval was granted by the La Trobe University Human Research Ethics Committee (ref: UHEC 15-088).

### 2.2. Study Design

**Figure 1:**
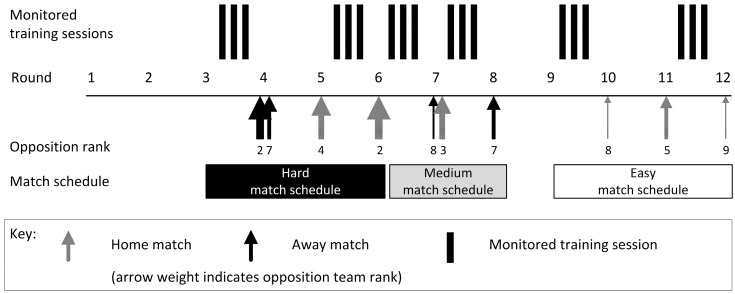
Schematic representation of data collection across the basketball season.

All players completed preliminary testing and were monitored over the course of a 17-round competitive basketball season. Six separate weeks of training data were collected during the competitive basketball season from three separate phases. Two weeks of training were monitored from each phase, where each week consisted of three team training sessions. The phases of monitored training were selected to represent periods of different match schedule difficulty, defined as easy, medium, and hard. The easy match schedule occurred between rounds 10 to 12, where the team played home matches against the two lowest ranked teams in the competition. The medium match schedule occurred between rounds 7 to 8, where the team played one double-header (i.e., two matches within a single round of competition) and one away match against moderately positioned teams. The hard match schedule occurred between rounds 4 to 6, where the team had an extensive travel schedule (away double-header) and a home match against the competition grand finalist (Figure 1). 

Preliminary testing included the measurement of body mass and standing stature according to the International Society for the Advancement of Kinanthropometry guidelines and procedures [20]. Additionally, a modified Yo-Yo intermittent recovery test (level 1; Yo-Yo-IR1), which included seven additional low speed stages prior to the commencement of the Yo-Yo-IR1, was completed. Movement speeds started at 3 km·h^−1^ and progressed by 1 km·h^−1^ for each stage until 9 km·h^−1^; after this, the original Yo-Yo-IR1 test was completed until exhaustion. Slower movement speeds occur frequently throughout basketball match-play [2,6], and inclusion of the slower movement speeds allowed for the calibration of relative exercise intensity across a broad range of movement speeds.

During all physical testing and training sessions the players wore a commercially available tri-axial accelerometer (Link; ActiGraph, Pensacola, FL, USA) on the upper-back as previously described [7,13], which recorded accelerations at 100 Hz. Previous research has established high levels of reliability for ActiGraph accelerometers [21,22,23]. In addition, breath-by-breath oxygen consumption (Oxycon Mobile, Jaeger, Germany) was recorded during the modified Yo-Yo-IR1 in order to establish individual relationships between accelerometry and $\dot{V}$O_2_.

### 2.3. Data Analyses

Accelerometer data were downloaded using the manufacturer’s software (ActiLife v12; ActiGraph, USA). Exercise intensity was quantified using AvF_Net_ as previously described [7,13]. To calculate AvF_Net_, the three planes of tri-axial accelerations were filtered using a dual-pass, fourth order Butterworth filter (high pass: 0.1 Hz, low pass: 15 Hz). These cut-off frequencies were chosen to remove gravity [24,25] and noise [26,27] components, respectively. After filtering, the product of the instantaneous acceleration vector and player’s body mass was used to determine instantaneous net force (F_Net_). The average F_Net_ (AvF_Net_) for user-selected periods was calculated in 1-s epochs using customised software (LabVIEW 2016; National Instruments, Austin, Texas, USA). In addition, interpolated $\dot{V}$O_2_ was included in the output from the modified Yo-Yo-IR1. To quantify the exercise dose for the entire training session, the numerical integral of AvF_Net_ and exercise duration was used to calculate accumulated impulse (Impulse), measured in Newton seconds (N·s).

Resting $\dot{V}$O_2_ was determined during 5-min seated rest prior to the beginning of the modified Yo-Yo-IR1. Accelerometer and $\dot{V}$O_2_ data were synchronised during the modified Yo-Yo-IR1 during the initial shuttle, where the acceleration signal was reconciled with the commencement of $\dot{V}$O_2_ recording. For all stages of the modified Yo-Yo-IR1 the acceleration signal was selected from the commencement of movement, which was identified as the moment when the resultant acceleration began to rise from rest, until the completion of the 40-m stage using the custom software to determine AvF_Net_ and $\dot{V}$O_2_. Peak $\dot{V}$O_2_ was determined as the greatest 5-s average $\dot{V}$O_2_ achieved during the final completed Yo-Yo-IR1 stage. $\dot{V}$O_2_ reserve ($\dot{V}$O_2_R) was calculated for each individual in order to represent relative maximum $\dot{V}$O_2_ above rest, by subtracting resting $\dot{V}$O_2_ from peak $\dot{V}$O_2_. Subsequently, AvF_Net_ and average $\dot{V}$O_2_R for each completed stage were correlated and best-fit linear relationships were generated for all players (r^2^ = 0.93–0.97). 

Accelerometry data from all recorded training sessions included all activity, stoppages, and time-outs beginning from the commencement of the warm-up to the completion of the final drill or cool-down. Training schedules included three separate training sessions per week (Sessions 1–3) with each training session consisting of warm-up drills, skill drills, offensive and defensive technical/tactical drills, and match-simulation drills.

Predicted $\dot{V}$O_2_R during training sessions were determined from AvF_Net_ (1-s epochs) using the player’s linear relationship developed from the Yo-Yo-IR1. Relative exercise intensity was categorised into seven intensity zones similar to those identified by the American College of Sports Medicine [28] being: sedentary behaviour (<20% $\dot{V}$O_2_R); very light (20–<30% $\dot{V}$O_2_R); light (30–<40% $\dot{V}$O_2_R); moderate (40–<60% $\dot{V}$O_2_R); vigorous (60–<90% $\dot{V}$O_2_R); maximal (90–<100% $\dot{V}$O_2_R); and supramaximal (≥100% $\dot{V}$O_2_R). Total time and proportion of time in all intensity zones were determined for all players across all training sessions. Outcome measures were calculated for all players and data were separated by playing position: front-court players (small forwards, power forwards, and centres; *n* = 5) and back-court players (point guards and shooting guards; *n* = 4).

### 2.4. Statistical Analyses

Statistical analyses were completed using IBM SPSS Statistics (v24; IBM Corporation, Armonk, NY, USA). Shapiro-Wilk tests confirmed that the assumption of normality was not violated, and group data were expressed as mean ± standard deviation (SD). Repeated measures two-way mixed model analyses of variance (ANOVA) (within factors: Match schedule and Session; between factor: Position) was used to determine the effect of match schedule difficulty, session, and position on exercise dose (Impulse) and intensity (AvF_Net_ and the proportion of time in all intensity zones). Effect sizes are presented as partial eta-squared statistic (ƞ^2^*_p_*). Mauchly’s test was consulted and Greenhouse–Geisser correction was applied if the assumption of sphericity was violated. Significant interactions or main effects were followed up with simple main effect analyses with pairwise comparisons using Bonferroni correction. Significance was set at *p* < 0.05.

## 3. Results

Average exercise intensity (AvF_Net_) across all 18 training sessions was 293 ± 40 N and was not different between match schedule, session, or playing position. The majority of activity during training was spent performing sedentary behaviour or very light intensity activity (64.3 ± 6.1%). Front-court position players performed a greater proportion of very light intensity activity during training sessions when compared with back-court players (mean difference: 6.8 ± 2.8%; Position effect: F_(1,7)_ = 5.798; *p* = 0.047; ƞ^2^*_p_* = 0.453). Back-court position players performed more supramaximal intensity activity when compared with front-court players during the medium match schedule (mean difference: 4.5 ± 1.0%; Match schedule × Position interaction: F_(2,14)_ = 9.323; *p* = 0.003; ƞ^2^*_p_* = 0.573). There were no positional differences in the proportion of time in all other intensity zones across all three match schedules (Table 1). 

The proportion of time performing very light intensity activity was different according to difficulty of match schedule (Match schedule effect: F_(2,14)_ = 4.761; *p* = 0.026; ƞ^2^*_p_* = 0.405), where more very light intensity activity tended to be performed during the easy match schedule compared with the hard match schedule (mean difference: 2.0 ± 2.0%; *p* = 0.062). Match schedule difficultly had no influence on the proportion of time in all other intensity zones. The proportion of vigorous intensity activity was different between sessions (Session effect: F_(2,14)_ = 5.271; *p* = 0.020; ƞ^2^*_p_* = 0.430). There was a greater proportion of vigorous intensity activity during Session 3 when compared with Session 1 (mean difference: 1.5 ± 1.2%; *p* = 0.026) through each match schedule.

**Figure 2:**
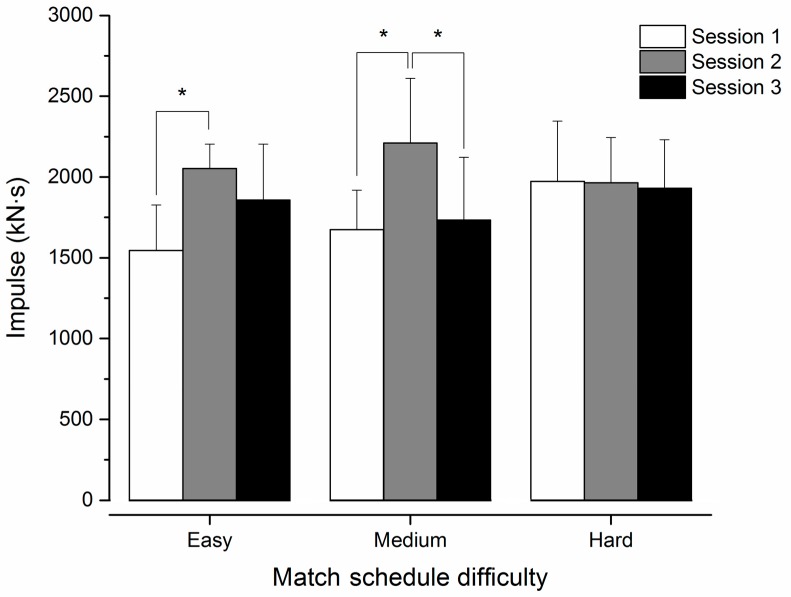
Exercise dose (Impulse) for easy, medium, and hard match schedules. Mean ± standard deviation. * Different between sessions (*p* < 0.05).

Mean exercise dose (Impulse) across all 18 training sessions was 1939 ± 258 kN·s. Playing position had no influence on the exercise dose received across match schedules (Position × Match schedule interaction: F_(2,14)_ = 0.133; *p* = 0.877; ƞ^2^*_p_* = 0.019) or training sessions (Position × Session interaction: F_(2,14)_ = 0.374; *p* = 0.695; ƞ^2^*_p_* = 0.051). The pattern of exercise dose during the three team training sessions per week changed according to the difficulty of match schedule (Figure 2; Match schedule x Session interaction: F_(4,28)_ = 4.224; *p* = 0.008; ƞ^2^*_p_* = 0.376). Exercise dose during Session 2 was greater when compared with Session 1 (mean difference: 537 ± 156 kN·s; *p* = 0.010) and Session 3 (mean difference: 476 ± 186 kN·s; *p* = 0.001) during the medium match schedule and greater compared with Session 1 (mean difference: 509 ± 107 kN·s; *p* = 0.01) during the easy match schedule. Exercise dose was similar between sessions during the hard match schedule (*p* ≥ 0.941).

## 4. Discussion

This is the first study to assess the relative exercise intensity of basketball training using a method that is suitable for sports involving rapid changes in movement patterns and intensities. The main findings of this study demonstrate that exercise dose varied between training sessions (i.e., Session 1, Session 2, Session 3) during easy and moderate difficulty match schedules but not during hard match schedules. Match schedule had no influence on the average exercise intensity and limited influence on the proportion of time spent in each intensity zone. Furthermore, few position-specific differences existed in the exercise dose, average exercise intensity, or the proportion of time spent in each intensity zone during training sessions completed by an elite women’s basketball team. 

The present study identified that the majority of exercise during basketball training sessions (64%) was spent performing either sedentary behaviour or very light intensity exercise. Results from a previous study, which used similar methods to the current study, show that a slightly lower proportion (approximately 59%) of match-play was spent performing either sedentary behaviour or very light intensity exercise [7]. Additionally, previous time-motion analyses have identified lower proportions (30–42%) of live match-play performing low-intensity and recovery activities (e.g., standing, walking) [6,9]. Taken together, these findings suggest that basketball training sessions are associated with greater periods of sedentary behaviour, likely due to inclusion of technical/tactical drills that involve large portions of time standing and walking while receiving coaching instruction [29]. Coaches should be aware that providing large amounts of instruction might compromise the match-specificity of training sessions. Therefore, technical/tactical drills can be combined with conditioning goals in order to more closely replicate match demands [30].

Despite large proportions of sedentary behaviour and very light intensity exercise during basketball, previous investigations consistently report high average physiological responses over the course of basketball training sessions. For example, mean $\dot{V}$O_2_ values during basketball training have been reported in the range of 60–80% $\dot{V}$O_2max_ [4,31]. Additionally, heart rate responses are typically in the range of 85–90% of maximum [31,32,33,34]. High physiological intensities during basketball have been reported because brief rest periods and active recovery (i.e., walking and jogging) during basketball are insufficient to permit full physiological recovery [12]. Thus, accelerated $\dot{V}$O_2_ kinetics at the onset of a work interval, in addition to cardiorespiratory lag, means that the physiological response at a particular point in time does not directly reflect the actual intensity of activity being undertaken. Therefore, physiological responses such as heart rate and $\dot{V}$O_2_ cannot truly reflect the exercise intensity during intermittent exercise, such as basketball. This highlights the importance of using accelerometry-derived AvF_Net_ to accurately quantify short duration bouts of intermittent exercise. This method could be important for athlete monitoring in order for coaches to replicate the most demanding aspects of match-play in training sessions, which can maximise training benefits and improve performance [35]. Additionally, findings from previous research suggest that periods of higher exercise dose throughout a basketball season are associated with greater risk of injury [36]. Therefore, monitoring accelerometry-derived AvF_Net_ throughout a basketball season might be useful to identify periods of heightened injury risk as a consequence of elevated exercise dose.

The present study identified that basketball training sessions elicit a similar accelerometry-derived exercise dose and proportion of time in each relative exercise intensity zone between front-court and back-court playing positions. This finding corroborates previous research, which found that movement demands during basketball training, assessed via time-motion analyses, were largely similar between playing positions [29]. Conversely, these findings are in direct contrast to the positional differences observed via time-motion analyses and physiological responses during basketball match-play [2,6,9,12]. Furthermore, recent evidence identified that these positional differences extend to accelerometry-derived relative exercise intensities during basketball match-play [7], suggesting that exercise dose and proportion of time in each exercise intensity zone during training is not always reflective of match-play. Similar exercise dose and intensity between playing positions during training sessions indicates that the individual positional demands of match-play are not replicated during training sessions. This might be due to logistical factors, such as a lack of time, space, and resources, which can make it difficult for coaches to individualise training for team sports. As such, during team training sessions, all players are often prescribed the same training drills. For this study, no feedback regarding players’ exercise dose or proportion of time in each intensity zone was provided to the coach, thus the exercise dose received is based solely upon the coach’s exercise prescription and the exercise completed by players. Therefore, it is possible that providing objective feedback of the exercise dose received by players and proportion of time in each intensity zone could assist coaching staff to calibrate exercise prescriptions and better replicate the exercise dose from match-play.

Both the exercise dose received by athletes and proportion of time in each intensity zone remained largely similar across the course of the season, despite variability in the difficulty of match schedule. Nevertheless, exercise dose varied between training sessions (i.e., Session 1, Session 2, Session 3) during the easy and medium match schedules. Increased exercise dose during Session 2 during the easy and moderate match schedules might be the coach’s attempt to compensate for the reduced exercise dose during the recovery session (Session 1) and pre-match tapering (Session 3). On the other hand, exercise dose did not vary between training sessions during the hard match schedule. These findings corroborate previous research from professional men’s basketball, which identified that exercise dose is related to the competition schedule [1]. Specifically, subjectively measured exercise dose (rating of perceived exertion × duration) from training sessions was also lower both pre- and post-match [1]. It is well-established that tapering can assist in improving competition performance [37]; however, future research should assess the most effective tapering strategy for the unique demands of basketball competition that often involve one or two competitive matches every week.

## 5. Conclusions

The exercise dose and intensity received by athletes remained largely similar throughout the competitive season despite variability in the difficulty of match schedule. Although coaches might be reducing exercise dose for recovery and pre-match tapering during easy and moderate difficulty match schedules, there was no evidence of training periodisation during hard match schedule. Furthermore, there were few position-specific differences in exercise dose and proportion of time in each intensity zone over the course of an elite women’s basketball season. Objective monitoring of the exercise dose in training and match-play via accelerometry-derived AvF_Net_ might enable coaches to better prescribe match-specific exercise during training.

## Figures and Tables

**Table 1:** Proportion of total duration (%) spent in each intensity zone for front-court and back-court players for each phase of the competitive season.

	Sedentary	Very Light	Light	Moderate	Vigorous	Maximal	Supra-Maximal
***Easy***							
front-court	38.5 ± 10.0	22.3 ± 5.6	12.1 ± 4.7	11.0 ± 3.3	11.5 ± 2.7	2.3 ± 1.0	2.2 ± 1.4
back-court	45.9 ± 7.6	16.4 ± 1.4	9.4 ± 2.9	10.2 ± 4.4	11.2 ± 1.2	3.3 ± 1.4	3.6 ± 1.7
***Medium***							
front-court	40.7 ± 13.1	21.6 ± 5.8	11.9 ± 4.9	10.3 ± 3.7	11.6 ± 3.8	2.4 ± 0.6	1.4 ± 0.5 *
back-court	52.2 ± 4.1	14.4 ± 1.0	7.2 ± 1.6	6.7 ± 1.6	9.9 ± 4.1	3.6 ± 1.4	5.9 ± 2.2
***Hard***							
front-court	43.8 ± 10.7	20.8 ± 5.2	10.9 ± 5.6	8.6 ± 2.4	12.0 ± 3.4	2.5 ± 0.9	1.3 ± 0.5
back-court	51.8 ± 6.4	14.4 ± 1.0	6.7 ± 2.0	6.8 ± 1.6	10.2 ± 4.7	3.5 ± 1.5	7.0 ± 5.8
***Total***							
front-court	40.8 ± 10.9	21.6 ± 5.4 *	11.7 ± 4.8	10.1 ± 2.8	11.7 ± 3.0	2.4 ± 0.6	1.7 ± 0.7
back-court	50.2 ± 4.4	14.8 ± 6.7	7.8 ± 1.8	7.8 ± 2.0	10.3 ± 3.2	3.5 ± 0.9	5.6 ± 2.7

Mean ± standard deviation. * Different to back-court (*p <* 0.05). $\dot{V}$O_2_R: Volume of oxygen uptake reserve. Sedentary: <20% $\dot{V}$O_2_R; Very Light: 20–<30% $\dot{V}$O_2_R; Light: 30–<40% $\dot{V}$O_2_R; Moderate: 40–<60% $\dot{V}$O_2_R; Vigorous: 60–<90% $\dot{V}$O_2_R; Maximal: 90–<100% $\dot{V}$O_2_R; Supramaximal: ≥100% $\dot{V}$O_2_R.
